# Writing Skills Development for Graduate Studies and Career Readiness in Science and Aging Fields: A Case Study Approach

**DOI:** 10.3389/fpubh.2021.727064

**Published:** 2021-12-02

**Authors:** Amanda Stafford McRell, Betty L. Wilson, Sue E. Levkoff

**Affiliations:** College of Social Work, University of South Carolina, Columbia, SC, United States

**Keywords:** writing skills, aging, science, racially diverse students, ethnically diverse students

## Abstract

Increasing the number of racially and ethnically underrepresented students who pursue scientific graduate studies in programs focusing on science and aging offers an opportunity to increase the number of aging specialists while simultaneously promoting diversity in the research labor market and supporting new ideas. This case study aims to better understand how students participating in an academic preparatory program experience a writing class contextualized within (1) students' writing background and (2) students' future ambitions related to science and aging. The individually-tailored writing class was taught as a critical component of a comprehensive educational program that targets underrepresented racial and ethnic minority undergraduate students who are interested in pursuing scientific graduate studies in fields related to aging. The researchers conducted semi-structured qualitative interviews with students (*n* = 4) enrolled in the 24-month fellowship training program, which included participation in the writing course during the summer prior to their senior year of undergraduate education. All participants were young adult college students who identified as Black or African American and female. Using thematic coding, statements about professional writing skills were divided into four primary themes: (1) prior experiences, (2) class experiences, (3) future goals and ambitions, and (4) structural considerations. These themes suggest potential implications for effective interventions aimed to advance the writing skills and academic and career readiness of racially and ethnically diverse students entering fields of science and aging.

## Introduction

According to United States (US) census projections, the US population is both “graying and browning;” that is, rapidly becoming older and more racially and ethnically diverse (1, 2). From 2000 to 2030, the number of older adults in the US is expected to increase from 35 million to over 72 million (3). By 2050, the population of Black older adults is projected to triple, while the population of Latinx older adults is expected to increase 11-fold (4). With older adults projected to comprise ~20% of the US population in the future, and new advancements in health and technology, there is a growing need for researchers, advanced practitioners and advanced degree-holders specializing in aging. In addition to the need for aging specialists in general, there is a need for more racial and ethnic diversity in aging specialization.

Increasing the number of science, technology, engineering, and mathematics (STEM) students who pursue scientific graduate studies in programs focusing on science and aging offers an opportunity to increase the number of aging specialists while simultaneously promoting new ideas and new perspectives. However, these opportunities are challenged by a poor fit between undergraduate student writing skills and expectations for graduate school entry. This mismatch is exacerbated by longstanding disparities in the public education system that contribute to racially and ethnically diverse students' underexposure to advanced-level writing curricula and to the undervaluation of different writing styles. Thus, exposure to a curriculum that provides such students with individually-tailored writing skills development can impact their readiness for graduate programs in science and aging and better prepare them for entry into a rapidly developing job market.

This case study aimed to better understand how students within an academic preparatory program experience a writing class building on (1) students' prior writing experiences and (2) students' future ambitions related to science and aging. The personalized writing class was taught as a critical component of a comprehensive educational program that targets racially and ethnically diverse undergraduate students who are interested in pursuing scientific graduate studies in fields related to aging. Study findings suggest potential implications for effective interventions aimed to advance the writing skills and academic and career readiness of racially and ethnically diverse students entering fields of science and aging.

## Background and Rationale

### Importance of Undergraduate Writing Skills Development

The need for quality writing skills in science-related fields, including aging, is becoming more crucial than ever before. Recently, there has been newfound attention on the importance of early writing skills development for students at the undergraduate level, particularly across science disciplines, as students who can demonstrate strong written communication skills are considered qualified candidates for graduate programs (5). While STEM candidates on the job market are required to have professional writing skills, science and technology high school and college students have been found to more likely experience difficulties with written communication (6). A study by Jang (6) found that 50% of college students in science and technology fields lacked basic levels of reading and writing. Jang (6) suggests that education programs in STEM fields can better prepare students for the changing job market by creating “a continuous cycle where students practice communicating in learning contexts and get frequent professional feedback from peers and educators using a peer and self-assessment for writing, speaking and collaboration” (p. 297).

For graduate programs in science, the significance of quality writing skills is clear: successful researchers, advanced practitioners and advanced degree-holders must be able to effectively communicate information with other researchers and practitioners as well as the general public (7). Scientific writing is also essential for scholarly activities such as publishing peer-reviewed journal articles, submitting abstracts for conference presentations, and completing grant proposals. These activities, in turn, prepare students to be competitive on the job market, empowering productive professionals and leaders in their fields. While there is strong expectation and need for students pursuing graduate programs in science and aging to be excellent writers, many students have not acquired sufficient skills to be able to write effectively in their respective fields by the end of their undergraduate studies. Consequently, the lack of writing skills might diminish the likelihood of the candidate's acceptance into their graduate school of choice. Even if accepted, students may feel less prepared for the “writing demands and other requirements of graduate education and professional careers” [(5), p. 1].

Many reasons exist for the lack of writing preparedness among undergraduate students. With pressing demands to cover course content and large grading loads, instructors rarely have time to teach writing skills or provide students with substantial feedback on papers to help improve their writing (5, 7). Because it is presumed that students learn basic writing skills during high school including knowledge of punctuation, grammar, sentence structure, and citations, some instructors may neglect to focus class time on writing development (8). However, for many racially and ethnically diverse students, the lack of writing preparedness is far more salient and complex.

### Disparities in Writing Skills Development

An overwhelming number of racially and ethnically diverse students graduate from high school unprepared for the writing demands and rigors of college education (9). Research suggests that African American students in particular are less likely to be academically prepared for college, with those from economically distressed communities being the least ready for college-level curricula (9, 10). The tremendous disparity in preparation for racially and ethnically diverse students, especially African American students, is often “centered on the deficiencies of students, families, and communities,” with little attention to institutional and social factors, including structural racism, exclusion, and poverty that influence college readiness (9). School factors such as poor access to college preparatory courses, funding, quality teachers, and supportive school counselors also impact students' preparedness for college (9).

There are discrepancies in the ways in which writing instruction is taught and measured across diverse student populations. According to Green (11), African American students are taught “to edit out, not edit, their Black English usage rhetorically to inform or enhance their academic writing” (p. 154). Unfortunately, racially and ethnically diverse students who struggle with “editing” out their unique linguistic differences in written assignments may face poor evaluations from teachers who operate from a Westernized perspective of writing that prioritizes dominant ideas about what constitutes “good” academic and professional writing (11). Despite perceptions of academic and professional writing skills as being racially and culturally biased (12), these perceptions remain the benchmarks by which many students are evaluated for admission into graduate school (13) and thereby deemed successful within graduate programs (8). Thus, there is need to equip racially and ethnically diverse students with the knowledge and skills to meet and exceed these standards, as well as to empower them to recognize unique cultural and linguistic differences in their writing.

### Bridging the Gap—The Significance of Historically Black Colleges and Universities (HBCUs)

HBCUs are unique sites for academic and professional achievement and cultural pride that have been significantly shaped by racism, discrimination, and social exclusion (11). HBCUs are shown to have welcoming and nurturing campus settings that provide opportunities for racially and ethnically diverse students, especially African American students, to excel academically (14–16). HBCUs already exist to enhance the academic and professional trajectory of racially and ethnically diverse students (15), while taking into account their cultural and linguistic differences (11). As such, HBCUs are uniquely positioned to help bridge the gap in writing skills development and preparation for this student population. Importantly, HBCUs can serve as a unique pathway to increase the number of qualified racially and ethnically diverse students who pursue scientific graduate studies in programs focusing on science and aging. Thus, there is need to implement effective programs in collaboration with HBCUs to enhance the writing skills of students and help develop their readiness both for matriculating into graduate programs in science and aging, as well as to achieve success in the growing job market of STEM and aging.

## Description

This section provides a brief description of the overall research education training program as well as the writing course component, and how they both aim to prepare students for graduate studies and career readiness in science and aging fields. Beginning in 2015, a flagship research university in a southeastern US state established an NIA-funded undergraduate research training program “to increase the number of qualified racially and ethnically diverse students who pursue scientific graduate studies in programs focusing on science and aging.” Based in a predominately white institution (PWI), this program to advance diversity in aging research collaborates with five HBCUs in the same state. HBCUs are ideal partners because they have a large number of undergraduate students who identify as Black or African American and who are majoring in medical, science, technology, engineering or mathematics (MSTEM) fields, and “who, through exposure to a research education program focusing on aging research, might choose to enter scientific careers committed to addressing complex biological, biomedical, behavior and clinical challenges that accompany aging.” Students who participate in the project gain mentored research experience by working in a research laboratory of a faculty member from the PWI research institution, along with co-mentoring from a faculty member from their HBCU, and attend didactic classes on the biology and social aspects of aging and experiential workshops led by faculty members at HBCUs and at the PWI research institution. Participating students (fellows) live on campus in student housing at the PWI research university for close proximity to their labs and classes during the summer research program. As part of their summer experience, fellows prepare a poster which they present at the end of the summer at the PWI's Annual Summer Research Conference. Fellows are encouraged to further disseminate their research through poster presentations at conferences after the summer workshops, with financial support from the program to attend professional meetings.

In the first few years of the program (2016–2018) the program offered formal coursework related to the biology and social aspects of aging, research in aging, and professional development. A number of our students needed specific writing skills development. Additionally, in 2018 fellows completing their second summer of research training, which takes place prior to their senior year of college, requested additional time and support to prepare personal statements for graduate and medical school applications. In response, program staff introduced the writing skills course in summer 2019 as a core component of the comprehensive institutional research education program. This writing skills course aims to prepare emerging aging researchers to write more effectively for individuals, groups, organizations, communities and colleagues and to improve writing skills needed for graduate program admissions, scholarship applications and other opportunities.

We hoped that students who actively participated in the course would improve their writing skills and be better prepared for advanced studies in STEM fields related to aging. Specific aims of the course are for students to: (1) increase their confidence related to professional writing; (2) organize written documents clearly and effectively; (3) substantiate arguments using appropriate evidence; (4) develop a clear, concise writing style; (5) produce effective academic, research and e-communication documents; and (6) adhere to strong ethical values related to writing and written communication.

The writing skills course is delivered through lectures and discussion. The primary method of instruction is interactive, with hands-on writing activities both in and out of class, coupled with critical feedback and review from classmates and the course instructor. The course instructor is a White female doctoral student who has experience teaching graduate-level writing students at the PWI research institution. At the conclusion of this course students will have completed two five-paragraph essays related to an aging topic of interest and one personal statement. These high-quality products can be adapted for graduate or medical school applications, fellowship or scholarship applications, and many other opportunities for professional advancement.

## Methods

This qualitative research study analyzed the individual experiences and perceptions of a small number of students participating in a professional writing course contextualized within (1) their participation in a comprehensive advancing diversity in aging research intervention program, (2) their prior educational and professional writing experiences, and (3) their future educational/professional ambitions. This focus is consistent with that promoted by Smith et al. (17) and Yin (18). Data were collected through semi-structured phone interviews from senior fellows (*n* = 4) who participated in the writing course during the second summer of the 2-year program. Similar to Ory et al. (19), the authors believe that the case study approach we have taken can contribute importantly to the development of other evidence-based programs and practices (17, 19, 20). Although *n* = 4 is a small sample size, the number of participants is appropriate for community case studies (18, 21–23). All study procedures were approved by the University of South Carolina Institutional Review Board.

Researchers developed a codebook using inductive thematic analysis and iteratively analyzed each transcript, revising the codebook until no new themes emerged. Transcript data were coded by the first and second authors using a process of first-cycle, second-cycle axial coding (24). Analyses were conducted in NVivo-12 and theme prevalence was determined using a conceptual cluster matrix table (25, 26). As the experts of their experiences, students can provide valuable information about their educational and professional experiences in an effort to improve their writing skills and academic and career readiness.

## Results

All participants were young adult college students who identified as Black or African American and female. Statements about professional writing skills were divided into four primary themes: (1) prior writing preparedness, (2) current writing preparedness, (3) writing goals and ambitions, and (4) structural considerations.

### Prior Experiences

Statements under the theme of “prior writing preparedness” describe situations that took place prior to participating in the summer writing class, such as high school and college coursework. Some students felt equipped to engage in graduate-level academic writing because they were well-prepared by high school and college classes. One student described doing well in high school with minimal effort but experienced a more rigorous writing environment with more critical feedback at her undergraduate institution:

“*For me, I feel like high school was super easy. I was in all the hardest classes, you didn't have to study for anything. So I got into college and I'm getting my paper slashed up. I had to study hard. Because now I always study like real hard, so I think I definitely got humbled freshman year, learning that this is like the big leagues now. It's not the same. Going from school to school, I think it's natural.”*

Another student also stated that her undergraduate institution prepared her well for college-level writing, but not for doctoral-level writing skills toward which she is working. Although the summer writing class was similar to writing classes she had taken at her undergraduate institution and she experienced some overlap in instruction, she still found the course useful:

“*I'm not one to say that I'm a strong writer. So all writing for me is crucial. So anytime that I can practice my writing skills and actually have someone read it, and actually give me feedback on what I need to work on, is great. So I do not mind the repetitiveness because my writing is not PhD-level, for example. It's like a college-level, which is where I was but I want it to be PhD level, so I didn't mind the repetition.”*

Other students felt that their high school and college learning experiences did not prepare them to engage in graduate-level academic writing skills. For example, one student described how her college English composition course was a positive experience but that the class was not completely focused on writing:

“*We wrote papers but it was only like, two and… we also did a lot more presentations, for example we had to create a poster or something like that for my English Comp as a grade instead of actually writing a paper.”*

Another student had a similar experience:

“*I learned a lot in [the professional development classes] because honestly even though I took English my first year of college, I think I learned more in my writing class over the summer than in my first year at college… my English class here wasn't a terrible class, I just felt like it wasn't as useful as the writing class I had last summer.”*

One student described how her experiences in an underfunded, racially-separated public education system influenced her writing skills training and the opportunities she was exposed to as a high school student:

“*I know particularly in my community… a lot of the Black schools didn't have the same things as the white schools. The white schools were private schools, people would pay to send their white kids to these private schools just so they wouldn't have to intermingle with the Black people in the community. Within the white schools they have a lot of money from the county that they receive, it goes to the white schools first and then it was like the leftovers, even though there was more of us than them…. So our books are old and half the time the computers don't work. It's just really frustrating and I feel like if I would have went to a private school I probably would have had a better chance. Even in high school I didn't have teachers that look like me. They were from different programs because our county couldn't really afford to pay teachers so we would get these mediocre teachers who are usually white or another race…. I feel like if I went to a different school I probably would have had a better chance. More exposure to different opportunities and stuff like that.”*

### Current Writing Preparedness

Statements under the theme of “current writing preparedness” reflected what happened during the summer writing class, such as the writing projects they completed, their feelings about writing, and their skills related to writing. Students revealed specific skills they learned through the summer writing course including writing clearer, writing stronger, engaging in scholarly debate, seeking and incorporating critical feedback, and improved confidence.

One student described how completing assignments allowed her to craft a scholarly argument, engage meaningfully with feedback and write clearly and concisely using simple language:

“*We had, I believe it was two essays and a personal statement. I believe. Both of them were persuasive variety, trying to prove a point and the personal statement was totally up to us. We had deadlines that we had to meet. Our writing teacher gave us really great feedback. She'd tells what we could have done better, what we done wrong, what we done right, what we need to include as far as content, grammar, punctuation, all of that stuff. We learned different types of writing and how to approach them and how to recognize those different types of writing. We also learned how to breakdown articles… and not to sound where we were trying to sound overly smart, but just enough so that the reader could understand what we're trying to say.”*

Every student mentioned the benefit of engaging with feedback from the instructor and/or their peers during the summer writing course. The following is a story about how the course impacted a student's perspective about critical feedback and writing skill confidence:

One student described feeling nervous to send an advisor her paper. This student felt “*a little- not uncomfortable-but just nervous, I just knew that paper was going to get sliced up, which it did. But that's just how it goes. But I wasn't uncomfortable, just nervous that someone was going to read my paper and analyze what I did and if I did it right and stuff like that.”* But after the summer writing course she felt more comfortable opening herself up to feedback: “*It made my nerves go away, because now I understand, okay, the paper is not going to come out perfect the first time you write it. So it made me stronger, because now I write what I can, or write what I think is best or whatever, and then I just send it off with no regrets. And if it comes back and it has questions or feedback or if she sliced it up, then I just read the feedback, or even with [the writing course instructor] reading my personal statement, when people give me feedback, it makes me think, ‘Okay, maybe that did sound weird, or that did sound awkward. I should have changed this around.' So now I'm more open to it, and not so afraid. I think before I was like, ‘Oh, I don't want them to think I can't write.' Everybody has a hard time writing, especially when it comes to, like, scientific writing….So I feel like that's the hardest thing for me now, to [receive] criticism, when I'm just like, okay, I'm here, it's for me.”*

When asked if she felt comfortable sharing her writing with other people, one student responded:

“*At first, I wasn't. But now I'm more open to share it with other people because I feel like I'm better at receiving feedback and how to incorporate in writing teams now, rather than how I was before.”*

Because the class was very small, fellows received individualized writing skills coaching with specific deadlines for submissions and resubmissions. One student described the class size as follows:

“*I think it was because of the class size and how productive it was. I guess when we have deadlines we're adamant about meeting those deadlines over the summer… we were actually writing things that we needed. It had [tips] to make our writing better.”*

### Writing Goals and Ambitions

Statements under the theme of “writing goals and ambitions” include students' descriptions of writing-related future goals and ambitions and ways in which writing will help them achieve those goals. Because completing a personal statement was one course requirement, this empowered students to meet the short-term goal of applying to graduate school programs. Two different students described the personal statement requirement as follows:

“*I like how they incorporated the writing class because as a rising senior at the time, I know that I needed to complete my graduate school application and just different things that gave an extra push to start off the academic year with.”*

“*If it wasn't for [the writing class and the professional development class] I wouldn't even have applied early to my programs because by me actually doing my personal statement and taking the GRE when I actually got to school in August I didn't feel overwhelmed like some of my other classmates. So, I was already steps ahead, more steps ahead than the others. So that was really good, I would say my senior year with the program, it was very beneficial.”*

Other students described ways in which the skills they learned in the writing skills course would support a variety of academic, research and professional long-term goals:

“*I'm going to need to write personal statements. I'm going to have to do dissertations, I'm going to have to write grants one day. I'm going to have to do all of these different things and [if] I don't know how to do professional writing. I'm not going to be able to do any of those.”*

“*I'm really trying to help [mentor] with this so I can get a publication before going into grad school,”* she described how she used writing skills to write the literature review for the manuscript she is writing with her mentor. She also described how the writing skills will be useful in graduate school*: “I have to be able to write a whole dissertation, with [research area]- it's just so big it has a lot of writing.”*

“*Writing is everything that a [healthcare provider] does. So, in class you learn document, document, document, which means you have to effectively, efficiently and in the most simplest way, write exactly what's wrong with an issue or a problem, something you've seen. You have to write down everything. If you don't know how to write it and get your point across in one or two sentences, then somebody else isn't going to have time to read a paragraph worth of things. So something that I learned in class that actually translates to what I'm doing now is getting your point across quickly, and then later you can elaborate on that point. But don't take seven sentences to say you walked down the street.”*

### Structural Considerations

Statements under the theme of “structural considerations” describe structural factors that influence their experience in the STEM scholars program both societally (e.g., at systematic levels) and personally. Most of the students mentioned that being an HBCU student at a PWI was a culture shock given their cultural upbringing and previous educational experiences.

The following is a story about how the campus environment and social norms of a PWI impacted a student's experience in the summer program:

“*When I got to [PWI-redacted] it was very different, it was very different. Because in [HBCU-redacted] everyone was really friendly, everybody is speaking even when they don't know each other. And you know [PWI-redacted] it was just very different, the atmosphere, when people walk, they just don't say, “Excuse me.” They just bump into you, they're not friendly. So, that was a shocker to me. I would speak and they would just look at me like I'm crazy.”*

The student also discussed feeling conflicted about attending a PWI for graduate school given her previous summer experience.

“*So now that I'm actually going to PWI for grad school, I don't know what to expect. I can code switch but I just feel like it's going to be very different because I'm a very friendly person.”*

Another student shared her experience of being an HBCU student at a PWI and feeling the pressure to not appear as a “stereotype” about her racial group while on campus.

“*Well, it was a culture shock for me. Only because I came from an all-black elementary, an all-black middle and high and I came through an HBCU, so everyone that I've ever known has looked like me. Then when I got on [PWI-redacted] campus and I saw all of the Caucasian people, I was a little shocked because it was like I didn't want to seem like a stereotype. Because what I was comfortable with doing, I didn't want to make other people uncomfortable with how I look and that was never a concern for me and over the summer it became one. The second summer it got easier because I already knew what to expect, but walking around on campus, it was a shock.”* However, the student felt the summer program and campus experience at a PWI exposed her to the realities of graduate education and the job market as a minority. “*I feel like it's kind of prepared me for it because I know that as you go higher in the rank, unfortunately there's not going to be a lot of people... I'm not going to see a lot of people that look like me and that within itself is intimidating. I feel like this experience that I had over the past two summers at [PWI-redacted] will help me get more comfortable with the idea that it's okay and that I am now part of the minority again, when, my entire life I felt like the majority.”*

All of the students mentioned that the underrepresentation of racially and ethnically diverse professionals and leaders in their respective fields influenced their decision to pursue graduate studies and careers in science and aging. One student described how the lack of African American (AA) female doctors in health care settings motivated her to pursue a graduate degree in public health.

“*I guess that it's just not diversity in science and in public health period. And that just makes me go harder with this public health degree because when I actually talked to the people this summer with my research, they feel better if they see people that they look like. And that thought would lead to when I go to doctors, I preferably want to see an African American woman doctor but it's almost where we just lack it.”*

All students described the importance of feeling comfortable. One student described how diversity in science and aging related fields can help increase patients' level comfort and the quality of service they receive.

“*And if you're talking to someone that looks like you, then I feel like you'll always feel a little more comfortable. And if there's no diversity, then they're not being given the opportunity or fair chance. Then it's like you're never going to get to see a difference, or even know if that would make a difference.”*

Another student described a similar perspective:

“*I feel like it would make people more comfortable to want to go into health care. I feel most comfortable if I actually see someone that looks like me because it's like a connection there; I feel like they will be very relatable.”*

## Discussion

For students who felt well-prepared by previous writing education experiences, the writing to advance diversity in aging research course elevated their writing skills to the next level: supporting advancement from competitive undergraduate-level writers, to competitive graduate-level writers. For students who described feeling under-prepared by the writing instruction they received in high school and college, this course provided instruction on basic skills including grammar and sentence structure, as well as more advanced professional writing skills. This case study suggests that the success of the writing course was due to the individualized instruction method, which relied heavily on instructor feedback and iterative coaching to improve student skills.

Through writing classes, students gained experience completing specific assignments and editing those assignments based on feedback and peer review. These assignments allowed students to gain the skills necessary to engage meaningfully with critical feedback, participate in a scholarly debate with peers and mentors, and write more clearly and concisely. Students also gained more confidence in their ability to write. This confidence, coupled with increased writing skills and willingness to engage in critical feedback, will support students as they apply to, and begin graduate school programs.

Students reported that participating in the advancing diversity for aging research writing class supported both their short-term and long-term goals. Because completing a personal statement for graduate school was a core course requirement, students were able to begin their senior year at their undergraduate institution more prepared to begin applying for higher education programs. Gaining writing skills, gaining confidence, and gaining willingness to engage with critical feedback will support a variety of long-term goals including collaborating on publishable academic manuscripts, securing scholarships, fellowships and grants, writing graduate or doctoral level theses, and successfully engaging in a variety of research and professional activities.

Finally, students reported that the lack of racially and ethnically diverse professionals in their fields significantly influenced their decision to pursue graduate studies and careers in science and aging. With demographics in the US shifting rapidly—becoming older and more racially and ethnically diverse (i.e., “graying and browning”) (1, 2)—students underscore the need for more representation of racially and ethnically diverse professionals in science and aging specializations. Increasing diversity in science and aging related fields yields opportunities to challenge longstanding disparities impacting diverse populations and promote innovative solutions for equitable, culturally responsive services.

### Limitations

Though this study provides important insights into the experiences of Black undergraduate students in a PWI-based academic preparatory program, it does not include the experience of other underrepresented minority groups. Future research is needed to understand and examine how the experiences of the students in the sample compare to students from various racial and ethnic minority groups enrolled in academic preparatory programs.

## Conclusions and Implications

Individually tailored professional writing instruction offers a unique opportunity to prepare racially and ethnically diverse students for successful entry into graduate school and a distinguished advanced academic trajectory. For students attending HBCUs who plan to apply to graduate-level programs at PWIs, professional writing instruction may bridge gaps for both students who feel prepared and for students who feel unprepared. For students who already feel prepared for advanced graduate study, this course provides an opportunity to review and sharpen basic skills, reinforcing the idea that anyone can become a stronger, clearer writer. The course also provides an opportunity to prepare for writing experiences in a more rigorous, graduate-level learning environment, such as giving and receiving critical feedback and engaging in a written scholarly debate. For students who feel unprepared for advanced graduate study, the course provides remedial instruction on basic skills and responsive, iterative feedback to improve writing confidence as well as writing skills.

Future studies seeking to implement an PWI-based academic preparatory program in partnership with HBCUs and other minority-serving institutions should take in consideration the historical contexts of these institutions, including the cultural experiences they provide to students. In addition, future research on the impact of a personalized writing course for racially and ethnically diverse students is needed to assess the effectiveness and validity of such preparatory course in increasing students' writing development and readiness for graduate school and professional careers in aging and related fields.

## Data Availability Statement

The datasets presented in this article are not readily available because the qualitative data generated for this article are unable to be sufficiently de-identified. Requests to access the datasets should be directed to as51@email.sc.edu.

## Ethics Statement

The studies involving human participants were reviewed and approved by University of South Carolina Institutional Review Board. Written informed consent for participation was not required for this study in accordance with the national legislation and the institutional requirements.

## Author Contributions

All authors listed have made a substantial, direct, and intellectual contribution to the work and approved it for publication.

## Funding

The research reported in this publication was supported by a grant from the U.S. National Institutes of Health: National Institute on Aging (R25AG050484).

## Conflict of Interest

The authors declare that the research was conducted in the absence of any commercial or financial relationships that could be construed as a potential conflict of interest.

## Publisher's Note

All claims expressed in this article are solely those of the authors and do not necessarily represent those of their affiliated organizations, or those of the publisher, the editors and the reviewers. Any product that may be evaluated in this article, or claim that may be made by its manufacturer, is not guaranteed or endorsed by the publisher.
